# Dietary Quality Changes According to the Preceding Maximum Weight: A Longitudinal Analysis in the PREDIMED-Plus Randomized Trial

**DOI:** 10.3390/nu12103023

**Published:** 2020-10-02

**Authors:** Cristina Bouzas, Maria del Mar Bibiloni, Silvia Garcia, David Mateos, Miguel Ángel Martínez-González, Jordi Salas-Salvadó, Dolores Corella, Helmut Schröder, J. Alfredo Martínez, Ángel M. Alonso-Gómez, Julia Wärnberg, Jesús Vioque, Dora Romaguera, José Lopez-Miranda, Ramon Estruch, Francisco J. Tinahones, José Lapetra, Luís Serra-Majem, Aurora Bueno-Cavanillas, Rafael M. Micó-Pérez, Xavier Pintó, Miguel Delgado-Rodríguez, María Ortíz-Ramos, Andreu Altés-Boronat, Bogdana L. Luca, Lidia Daimiel, Emilio Ros, Carmen Sayon-Orea, Nerea Becerra-Tomás, Ignacio Manuel Gimenez-Alba, Olga Castañer, Itziar Abete, Lucas Tojal-Sierra, Jéssica Pérez-López, Andrea Bernabé-Casanova, Marian Martin-Padillo, Antonio Garcia-Rios, Sara Castro-Barquero, José Carlos Fernández-García, José Manuel Santos-Lozano, Cesar I. Fernandez-Lazaro, Pablo Hernández-Alonso, Carmen Saiz, Maria Dolors Zomeño, Maria Angeles Zulet, Maria C. Belló-Mora, F. Javier Basterra-Gortari, Silvia Canudas, Albert Goday, Josep A. Tur

**Affiliations:** 1 CIBER Fisiopatología de la Obesidad y Nutrición (CIBEROBN), Instituto de Salud Carlos III (ISCIII), 28029 Madrid, Spain; cristinabouvel@gmail.com (C.B.); mar.bibiloni@uib.es (M.d.M.B.); sgh950421@gmail.com (S.G.); davidfrom13@gmail.com (D.M.); mamartinez@unav.es (M.Á.M.-G.); jordi.salas@urv.cat (J.S.-S.); dolores.corella@uv.es (D.C.); jalfredo.martinez@imdea.org (J.A.M.); angelmago13@gmail.com (Á.M.A.-G.); jwarnberg@uma.es (J.W.); mariaadoracion.romaguera@ssib.es (D.R.); jlopezmir@gmail.com (J.L.-M.); RESTRUCH@clinic.cat (R.E.); fjtinahones@uma.es (F.J.T.); joselapetra543@gmail.com (J.L.); lluis.serra@ulpgc.es (L.S.-M.); xpinto@bellvitgehospital.cat (X.P.); eros@clinic.cat (E.R.); nerea.becerra@urv.cat (N.B.-T.); i.gimenez.alba@valencia.edu (I.M.G.-A.); ocastaner@imim.es (O.C.); iabetego@unav.es (I.A.); lutojal@hotmail.com (L.T.-S.); jessicaperezlopez@uma.es (J.P.-L.); angarios2004@yahoo.es (A.G.-R.); sacastro@clinic.cat (S.C.-B.); jc.fernandez@uma.es (J.C.F.-G.); josemanuel.santos.lozano@gmail.com (J.M.S.-L.); pablo1280@gmail.com (P.H.-A.); carmen.saiz@uv.es (C.S.); mzomeno@imim.es (M.D.Z.); mazulet@unav.es (M.A.Z.); marujabello@gmail.com (M.C.B.-M.); silvia.canudas@iispv.cat (S.C.); Agoday@parcdesalutmar.cat (A.G.); 2 Research Group on Community Nutrition & Oxidative Stress, University of Balearic Islands, & CIBEROBN, Guillem Colom Bldg, Campus E, 07122 Palma de Mallorca, Spain; 3 Health Research Institute of the Balearic Islands (IdISBa), 07120 Palma de Mallorca, Spain; marian.martin@gmail.com; 4 Department of Preventive Medicine and Public Health, IDISNA, University of Navarra, 31008 Pamplona, Spain; mdelgado@ujaen.es (M.D.-R.); msayon@unav.es (C.S.-O.); cflazaro@unav.es (C.I.F.-L.); javierbasterra@hotmail.com (F.J.B.-G.); 5 Department of Nutrition, Harvard T. H. Chan School of Public Health, Boston, MA 02115, USA; 6 Human Nutrition Unit, Biochemistry and Biotechnology Department, IISPV, Hospital Universitari de Sant Joan, Universitat Rovira i Virgili, 43201 Reus, Spain; 7 Department of Preventive Medicine, University of Valencia, 46100 Valencia, Spain; 8 Unit of Cardiovascular Risk and Nutrition, Institut Hospital del Mar de Investigaciones Médicas Municipal d’Investigació Mèdica (IMIM), 08003 Barcelona, Spain; hschroder@imim.es; 9 CIBER Epidemiología y Salud Pública (CIBERESP), Instituto de Salud Carlos III (ISCIII), 28029 Madrid, Spain; vioque@umh.es; 10 Cardiometabolic Precision Nutrition Program, IMDEA Food, CEI UAM + CSIC, 28049 Madrid, Spain; 11 Department of Nutrition, Food Sciences, and Physiology, Center for Nutrition Research, University of Navarra, 31008 Pamplona, Spain; 12 Bioaraba Health Research Institute, Osakidetza Basque Health Service, Araba University Hospital, University of the Basque Country UPV/EHU, 48013 Vitoria-Gasteiz, Spain; 13 Department of Nursing, School of Health Sciences, University of Málaga-IBIMA, 29071 Málaga, Spain; 14 Instituto de Investigación Sanitaria y Biomédica de Alicante, ISABIAL-UMH, Miguel Hernández University, 46020 Alicante, Spain; abueno@ugr.es; 15 Lipids and Atherosclerosis Unit, Department of Internal Medicine, Maimonides Biomedical Research Institute of Cordoba (IMIBIC), Reina Sofia University Hospital, University of Cordoba, 14004 Córdoba, Spain; 16 Department of Internal Medicine, IDIBAPS, Hospital Clinic, University of Barcelona, 08036 Barcelona, Spain; 17 Department of Endocrinology, Virgen de la Victoria Hospital, Instituto de Investigación Biomédica de Málaga, IBIMA, University of Málaga, 29010 Málaga, Spain; 18 Department of Family Medicine, Research Unit, Distrito Sanitario Atención Primaria Sevilla, 41013 Sevilla, Spain; 19 Institute for Biomedical Research, University of Las Palmas de Gran Canaria, 35016 Las Palmas, Spain; 20 Department of Preventive Medicine, University of Granada, 18071 Granada, Spain; 21 Fundación Semergen, 28009 Madrid, Spain; socochato68@gmail.com; 22 Cátedra de Investigación en Cronicidad, Miguel Hernández University-Semergen, 03550 Sant Joan d’Alacant, Spain; 23 Lipids and Vascular Risk Unit, Internal Medicine, Hospital Universitario de Bellvitge, Hospitalet de Llobregat, 08907 Barcelona, Spain; 24 Department of Health Sciences, Center for Advanced Studies in Olive Grove and Olive Oils, University of Jaen, 23071 Jaen, Spain; 25 Department of Endocrinology and Nutrition, Instituto de Investigación Sanitaria Hospital Clínico San Carlos (IdISSC), 28040 Madrid, Spain; maria.ortiz.929@gmail.com; 26 Department of Endocrinology, IDIBAPS, Hospital Clinic, University of Barcelona, 08036 Barcelona, Spain; aaltesboronat@yahoo.com; 27 Department of Endocrinology, Fundación Jiménez-Díaz, 28040 Madrid, Spain; bogdanaluca@yahoo.com; 28 Nutritional Control of the Epigenome Group, Precision Nutrition and Obesity Program, IMDEA Food, CEI UAM + CSIC, 28049 Madrid, Spain; lidia.daimiel@imdea.org; 29 Department of Endocrinology and Nutrition, Lipid Clinic Unit, Institut d’Investigacions Biomèdiques August Pi Sunyer (IDIBAPS), Hospital Clínic, 08036 Barcelona, Spain; 30 Servicio Navarro de Salud, Osasunbidea, IDISNA, 31003 Pamplona, Spain; 31 Centro Salud Raval, 03203 Elche-Alicante, Spain; andrea.bernabe.casanova@gmail.com

**Keywords:** body image, dietary pattern, maximum weight, Mediterranean diet, PREDIMED-Plus

## Abstract

One-year dietary quality change according to the preceding maximum weight in a lifestyle intervention program (PREDIMED-Plus trial, 55–75-year-old overweight or obese adults; *n* = 5695) was assessed. A validated food frequency questionnaire was used to assess dietary intake. A total of 3 groups were made according to the difference between baseline measured weight and lifetime maximum reported weight: (a) participants entering the study at their maximum weight, (b) moderate weight loss maintainers (WLM), and (c) large WLM. Data were analyzed by General Linear Model. All participants improved average lifestyle. Participants entering the study at their maximum weight were the most susceptible to improve significantly their dietary quality, assessed by adherence to Mediterranean diet, DII and both healthful and unhealthful provegetarian patterns. People at maximum weight are the most benefitted in the short term by a weight management program. Long term weight loss efforts may also reduce the effect of a weight management program.

## 1. Introduction

Overweight and obesity, understood as an excess of body fat, are associated to an increased risk of several diseases [1], which might reduce quality of life and increase mortality [1]. Overall, prevalence of non-transmissible chronic diseases among individuals increase after 55 years, especially those related to an excess of body weight or those prone to aggravate by an excess of body weight [2].

The PREDIMED (PREvención con DIeta MEDiterránea) study has found that harmful effects of metabolic syndrome on cardiovascular health occurs less often when adherence to the Mediterranean diet (MedDiet) is high [3]. Lately, the PREDIMED-Plus study has proved that higher adherence to a MedDiet improved nutritional density [4], as well as weight loss in the first year of treatment [5,6]. These results support this intervention as a proper weight management and disease prevention strategy [7].

Observational studies have related continued healthy habits adherence to improvement of long-term outcomes such as weight loss [8]. However, what makes an overweight person pursuit weight loss? On the one hand, history of obesity has been related to a higher spontaneous weight loss [9]. Nevertheless, unintentional weight loses have been related to more unfavorable health behaviors than intentional weight losses, which are related to morbidity and mortality [10]. On the other hand, rather than the own body weight, perceptions are more likely to boost weight management actions [11] as illustrated by Higgins’ regulatory focus theory [12]. Unfortunately, aging has been associated to lower overweight perception and lower weight concerns [1,11]. This might negatively affect health, due to ignoring the overweight condition and its implications [1,11].

Therefore, the aim of the present study was to assess 1-year dietary quality changes according to the reported preceding maximum weight in the multicenter, randomized, primary-prevention trial (PREDIMED-Plus) that is based on an intensive lifestyle intervention program.

## 2. Materials and Methods

### 2.1. Study Design

This research is a prospective cohort analysis of baseline and 1-year data within the frame of the PREDIMED-Plus trial, an ongoing 6-year parallel-group, multicenter, randomized trial of combined physical activity and dietary intervention for cardiovascular disease morbimortality prevention in overweight and obese individuals, conducted in 23 Spanish recruiting centers (universities, hospitals and research institutes). Briefly, the trial compares between two interventions: (1) an energy reduced MedDiet with physical activity promotion and an intensive behavioral support, versus (2) usual care consisting of energy unrestricted (ad libitum) MedDiet with less intensive behavioral support and no physical activity recommendations. The first group aims to lose weight, while the usual care group does not. Further details on the study protocol can be found elsewhere [7] and at http://predimedplus.com/. The trial was registered in 2014 at the International Standard Randomized Controlled Trial (ISRCT; http://www.isrctn.com/ISRCTN89898870) with number 89898870.

### 2.2. Participants, Recruitment, Randomization, and Ethics

Community-dwelling adults were eligible if they were aged 55–75 (60–75 for women). Overweight or obesity was required (body mass index (BMI) between 27 and 40 kg/m^2^), as well as meeting at least 3 metabolic syndrome criteria according to the updated harmonized definition of the International Diabetes Federation and the American Heart Association and National Heart, Lung and Blood Institute [13]. Exclusion criteria for the present study were reported elsewhere [7].

**Figure 1:**
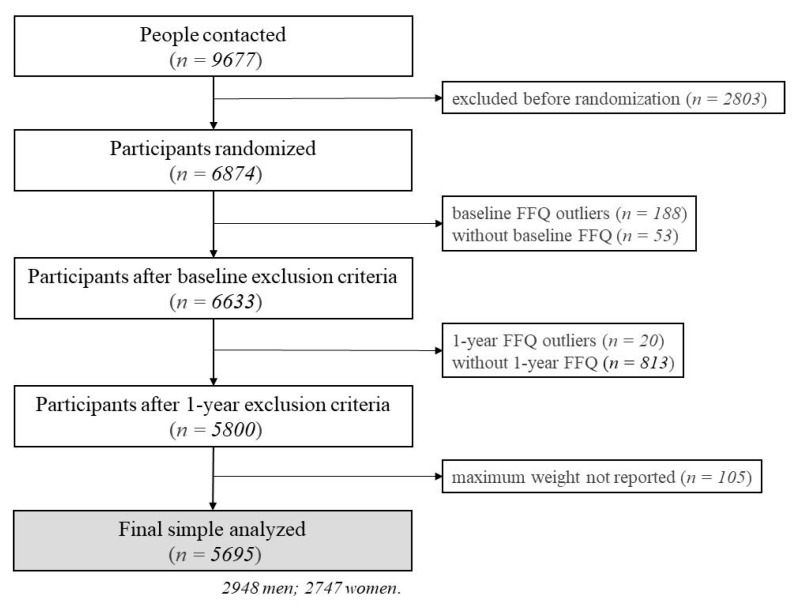
Flow-chart of the study participants. FFQ: food frequency questionnaire.

A total of 9677 people were contacted, from 5 September 2013 to 31 October 2016. Of these, 6874 participants were eligible for the study and were randomized into one of the two groups, in a 1:1 ratio. Randomization was stratified by center, sex, and age categories. When both members of a couple were living in the same household, they were randomized as a cluster (Figure 1).

All institutions participating approved the procedures and study protocol according to Declaration of Helsinki’s ethical standards. The study protocols followed the Declaration of Helsinki ethical standards and were approved by the Ethics Committee of Research of Balearic Islands (ref. CEIC-IB2251/14PI). All participants provided written informed consent.

### 2.3. Dietary Assessment

Dietary intake was assessed by registered dietitians at baseline and at 1-year follow up. A semi quantitative 143-item food frequency questionnaire (FFQ) previously validated for the Spanish population [14] was used for that purpose. A regular portion size was established for each item, and nine consumption frequencies were available, ranging from “never or almost never” to “≥6 times/day”. Nutrient and energy intakes were calculated multiplying the obtained frequency by nutrient or energy composition of the specified portion size for each food item. This was done with the support of a computer program based on information in Spanish food composition tables [15,16]. Dietary supplements declared in the FFQ were kept in mind for micronutrient intake assessment.

Participants reporting extreme total energy intakes (<500 or >3500 kcal/day in women or <800 or >4000 kcal/day in men) were excluded from the analysis [17]. For this reason, at baseline, 241 subjects were excluded (53 incomplete FFQ and 188 reporting extreme total energy intakes). Of the remaining, 833 were excluded at 1 year follow up (813 incomplete FFQ and 20 reporting extreme total energy intakes). Therefore, the sample size reduced up to 5800 participants (Figure 1).

Macro and micronutrient intake in the present study are expressed as nutritional density. This was the result obtained from dividing everyone’s intake of a given nutrient by the amount of calorie intake reported by that individual. This was done to avoid bias produced by the inter-individuals’ variability of energy intake.

### 2.4. Determination of the Dietary Indexes

Three different dietary indexes were determined. Dietary inflammatory index was related to micronutrient intake. 17-item MedDiet index and Provegetarian pattern indexes (healthful and unhealthful) related to food intake. The 17-item MedDiet index was closely related to the intervention. Hence authors also decided to assess food intake with a different dietary index. Provegetarian pattern indexes were chosen because the advice provided to participants in the study recommended a MedDiet [7]. The MedDiet is a plant-based diet [3]. However, not all plant-derived foods are healthy [18]. High adherences to a healthy provegetarian diet were associated to a reduced risk of overweight and obesity [18]. Together, the healthful and unhealthful provegetarian patterns provide an appropriate assessment of food patterns for the present study.

#### 2.4.1. Determination of the Dietary Inflammatory Index

Shivappa et al. [19] described the Dietary inflammatory index (DII) as an effective tool to assess inflammatory potential of the diet. The DII is based on a literature review and reports the effect of 45 nutrients, foods, and other dietary bioactive compounds on 6 inflammatory biomarkers (C-Reactive Protein, Tumor Necrosis Factor-alpha, and 4 interleukins: IL-1 β, IL-4, IL-6, IL-10). Positive DII is associated to a pro-inflammatory diet while negative scores are related to anti-inflammatory diets [19]. Methods to obtain DII have been previously described [19,20]. Briefly, each food item was assigned an overall inflammatory effect score. Standard mean intake of each parameter was subtracted from the individual’s intake of each parameter, and the result was divided by its standard deviation (SD). These values were converted to a centered percentile score and then multiplied by their overall food-parameter inflammatory effect score. DII score is the sum of all food parameters.

In the current study, FFQ questionnaire was the tool used to assess food parameters intake. Of the 45 food parameters, 15 could not be measured by the FFQ and were not included in the assessment of DII, as it was previously performed when food parameters were unavailable [20]. Thus, food parameters included were energy, carbohydrates, proteins, total fat, polyunsaturated fatty acids (PUFA), monounsaturated fatty acids (MUFA), saturated fatty acids (SFA), trans-fat, n-3 fatty acids, n-6 fatty acids, cholesterol, fiber, vitamin A, thiamin, riboflavin, niacin, vitamin B6, vitamin B12, folic acid, vitamin C, vitamin D, vitamin E, magnesium, iron, selenium, zinc, alcohol, garlic, green/black tea, and onion.

#### 2.4.2. Assessment of the Provegetarian Dietary Patterns (Healthful and Unhealthful)

Provegetarian dietary patterns were calculated according to Gómez-Donoso et al. [18]. Foods were divided into animal foods (dairy; eggs; meat; fish and seafood; animal fat; and miscellaneous food such as pizza, dressings, dry soups, etc.), healthy plant-foods (vegetables, fruits, legumes, whole grains, nuts, olive oil, tea, and coffee) and less-healthy plant-foods (refined grains, potatoes, sweets, desserts, fruit juices, and sugary beverages) [18]. Food consumption was adjusted by total energy intake through the residual method, separately for men and women [21]. The residuals or energy-adjusted estimates were ranked into quintiles. For healthful provegetarian food pattern assessment, positives scores were attributed to healthy plant food quintiles, and reverse scores were attributed to less-healthy plant food and animal food quintiles. For unhealthful provegetarian food pattern assessment, positives scores were attributed to less-healthy plant food quintiles, and reverse scores to healthy plant food and animal foods quintiles. Both healthful and unhealthful provegetarian food patterns could range from 18 to 90 (90 was highest adherence).

#### 2.4.3. Assessment of Adherence to Mediterranean Dietary Pattern

To assess MedDiet adherence, registered dietitians administered the 17-item MedDiet questionnaire [7], which is a modified version of the validated questionnaire used in the PREDIMED trial [7]. Each of the 17 items related to a food habit. Compliance with each food item scored 1, otherwise scored 0. Accordingly, the 17-item MedDiet questionnaire ranged between 0 and 17.

### 2.5. Body Image Assessment

An eating disorder questionnaire [7] was administered at baseline. Through that questionnaire, by the item “What has been your maximum weight in adult life??” participants reported their lifetime maximum ever reached weight (expressed in kg). The period of pregnancy was excluded, by asking women to report their maximum weight excluding pregnancy. A total of 105 participants did not report maximum weight and were excluded from the analysis. As a result, final sample size was of 5695 (2948 men and 2747 women) (Figure 1). The questionnaire additionally included other questions such as: “how old were you when you were at your maximum weight?” or “What would be your ideal weight right now?” Hence, participants reported their age when they registered their maximum weight and their reported ideal weight at the time of the interview.

Registered and trained dietitians measured height and weight in duplicate with a wall-mounted stadiometer and a high-quality electronic calibrated scale, respectively. Height was measured according to WHO standards [7]. BMI (current, maximum, and ideal) were calculated as weight in kilograms divided by the square of height in meters. BMI was categorized according to guidelines [22].

For this study, body image was expressed as difference between measured current weight and lifetime maximum reported weight. Current weight was the maximum weight in two circumstances. Firstly, when participants reported maximum weight fell within the range of ±2 kg of their current measured weight [23,24]; secondly, when participants reported lower maximum weight than measured current weight.

Subjects were categorized into one of the following three groups. (i) Participants currently at their maximum weight (*n* = 2181), (ii) participants who lost weight from their maximum weight but within the same BMI category (*n* = 1688), (iii) participants who lost weight from their maximum weight, and due to that weight loss they managed to descend their BMI category (*n* = 1826).

One of the exclusion criteria for the current study was reporting high weight loss in the 6 months previous entering the study [7]. Therefore, it could be assumed that those participants reporting lower current weight than maximum weight are successful weight loss maintainers (WLM), or in other words, the aforementioned groups respectively represent (i) participants entering the study at their maximum weight (*n* = 2181), (ii) moderate WLM (BMI decrease within the same category) (*n* = 1688), and (iii) large WLM (decrease of BMI category) (*n* = 1826).

### 2.6. Other Health Variables

Medical history and current medication were obtained. Blood pressure was measured in triplicate with a validated semi-automatic oscillometer (Omron HEM-705CP, Lake Forest, IL, USA) in a seated position. The three measures were taken after 5 min sitting rest, waiting for one minute between each take. The arm chosen to do the measures was the arm registering the highest diastolic blood pressure in the first visit of the run-in period. The arm chosen in everyone was not changed during the study. Cuff was strictly adjusted to the circumference of the upper arm. Overnight fasting (at least 8h) blood collections were analyzed in local laboratories. Biochemical analyses using overnight fasting blood samples (triglycerides, total cholesterol, HDL-cholesterol, and fasting plasma glucose) were performed by standard enzymatic methods. Further information on applied methods is available [7]. Abdominal obesity was assessed by measuring waist circumference in duplicate using an anthropometric tape, halfway between the last rib and the iliac crest.

Sedentary behaviors and physical activity were assessed by the validated Spanish version of the nurses’ health study questionnaire [25] and the validated Minnesota-REGICOR short physical activity questionnaire [26] respectively.

### 2.7. Statistical Analyses

Analyses were performed with the SPSS statistical software package version 25.0 (SPSS Inc., Chicago, IL, USA). All data are shown as mean and standard deviation (SD) except for prevalence, which is shown as sample size and percentage. Differences among groups for baseline descriptive characteristics were tested with one-way ANOVA, with Bonferroni’s post hoc analysis. Differences in prevalence among groups were tested using χ^2^ (all *p* values are two-tailed). 

Changes during 1-year in nutrients, foods, dietary patterns, physical activity, and BMI according to the three groups above described were analyzed by the Generalized Linear Model (GLM). The effect of the interaction was examined by using repeated-measures ANCOVA with 2 factors: time (baseline vs. 1 year) as repeated measure, group (3 groups abovementioned) and their interactions, with sex and intervention group as covariates. Because energy intake and physical activity were not involved in the assessment of DII and food variables, they were considered covariates (both as continuous variables) in the analysis of DII and food variables. The Bonferroni post hoc test was conducted to compare differences in the effects of each group within and between groups. Results were considered statistically significant if *p*-value (2 tailed) <0.05. Similar secondary analysis adjusting in addition by presence of type 2 diabetes mellitus (T2DM) at baseline was run, and it is represented in the same tables.

## 3. Results

Table 1 shows baseline characteristics of the participants. The group that managed to reduce their BMI category (large WLM) experienced an average BMI loss of 3.5 kg/m^2^; the group reducing weight within the same BMI category (moderate WLM) experienced an average loss of 1.7 kg/m^2^; in contrast, the group at their maximum weight did not experienced any weight loss. Age and ideal BMI were similar among groups. Moderate WLM group registered lower current and perceived BMI than the other two groups. Nonetheless, large WLM group (maximum BMI: 36.2 kg/m^2^, 55 years), reported higher maximum BMIs and at a younger age than those participants at their maximum weight (maximum BMI: 32.6 kg/m^2^, 61.6 years). Sex, intervention group and marital status were not distributed similarly among the three groups. Education level was higher among the group at their maximum weight at baseline. There were no differences in smoking habit among groups. Regarding metabolic syndrome components, no differences were found among groups for high blood pressure, low HDL-cholesterol, or abdominal obesity; however, the group at their maximum weight at baseline had the highest prevalence of hypertriglyceridemia (57.9% vs. 56.4% of the moderate WLM and 53.3% of the large WLM; *p* = 0.014) but the lowest rates of hyperglycemia (72% vs. 75% of the moderate WLM and 80% of the large WLM; *p* < 0.001). The proportion of hyperglycemia was higher in participants who had been in a higher BMI category prior to the inclusion.

Supplementary Table S1 presents intakes of macro- and micronutrients (expressed as nutritional density) at baseline and at 1-year time. Comparing changes among groups, the large WLM group reported the lowest increases through time for protein and fiber intake, as well as the highest increases in the intakes of PUFA and MUFA and the highest decrease in the intake of cholesterol. This group also reported the highest intakes of proteins and fiber among groups at baseline and 1-year, and cholesterol at baseline. However, baseline MUFA intake in them was lower than in the group at their current maximum weight. Participants in the large WLM group also reported significantly higher intakes of all vitamins and minerals; however, in comparison with the other groups, they reported the lowest increases through time. Vitamin E is the only exception, as the lowest intake increase was registered in the moderate WLM group. For vitamin B2 and vitamin D no changes time*group were observed. Finally, intake of carbohydrates was lower at baseline among those participants at their current weight, but intakes of total fat and trans-fatty acids were higher in this group, as well as the intake of SFA at 1-year.

Dietary items intakes at baseline and at one-year follow up are shown in Supplementary Table S2; however, most relevant results will be highlighted hereafter. As a result of the intervention, intake of all dietary items for all groups (within the group) changed from baseline to one year, with exceptions for coffee and tea for all groups and seafood, potatoes, and eggs for some groups. Comparing changes among groups, the highest increases through time were found in the group at their maximum weight for fruits (change: maximum weight: 52.3 g/day; moderate WLM: 38.9 g/day; large WLM: 38.5 g/day), vegetables (change: maximum weight: 41.9 g/day; moderate WLM: 32.4 g/day; large WLM: 27.4 g/day), and white meat items (change: maximum weight: 8.2 g/day; moderate WLM: 5.1 g/day; large WLM: 5.3 g/day). The same tendency was identified for blue fish (change: maximum weight: 6.6 g/day; moderate WLM: 5.5 g/day; large WLM: 5.0 g/day, *p* = 0.062). A lower intake at baseline of those foods can be observed for the above-mentioned group while at 1-year time all groups reported a similar consumption. However, time*group significances for fruits and blue fish are lost after adjustment by T2DM baseline prevalence. Nuts consumption at baseline was similar among groups; however, groups that managed to reduce their BMI category reported a higher increase in nuts consumption (change: maximum weight: 13.3 g/day; moderate WLM: 13.2 g/day; large WLM: 15.9 g/day). Milk and dairy consumption decreased through time. Those who reduced their BMI category registered the highest decrease but also reported the highest dairy intakes at baseline and 1 year. In some food groups no changes time*group were found; however, daily intake was significantly different among groups either at baseline or at 1-year time. Participants at their maximum weight at baseline reported higher consumption of red meat at baseline, convenience foods at one year and olive oil and fermented alcoholic beverages at baseline and 1 year; as opposed to a lower consumption of whole grains at baseline and legumes at baseline and one year compared to those far from their maximum weight.

Table 2 shows changes through time in BMI, physical activity, energy intake and dietary patterns. As a result of the intervention, BMI decreased in all groups. BMI decrease for one year was inversely proportional to weight loss previously achieved (change in BMI: maximum weight: −1.0 kg/m^2^; moderate WLM: −0.8 kg/m^2^; large WLM: −0.7 kg/m^2^). While total physical activity at baseline was higher in participants who were not at their maximum weight, all groups increased their physical activity because of the intervention, resulting in no differences in total physical activity at one year. Moderate and intense physical activities were responsible for the overall increase. No differences time*group were found for physical activity variables.

The highest energy intakes at baseline and at 1 year were registered in the group at their maximum weight; however, no differences in time*group were found for energy intake. All groups reduced similarly their energy intake after one year. Regarding dietary patterns, some relevant results ought to be highlighted. MedDiet adherence at baseline augmented proportionally to weight loss. As a result of the intervention, MedDiet adherence increased in all groups, but the biggest increase can be seen in the group entering the study at their maximum weight (change in adherence: maximum weight: 3.4; moderate WLM: 3.2; large WLM: 3.1). However, MedDiet adherence at one year was similar among groups. After adjustment by DMT2 baseline prevalence, increases in adherence to MedDiet were similar for all groups. Concerning the provegetarian patterns analyzed, no differences were found for healthy provegetarian pattern conversely to unhealthy provegetarian pattern. Baseline unhealthy provegetarian pattern was inversely proportional to weight loss. Nonetheless, the group at their maximum weight at baseline was the only group able to reduce the unhealthful provegetarian pattern from baseline to one-year follow-up (change: maximum weight: −0.7; moderate WLM: 0.3; large WLM: 0.2). Lastly, the DII analysis showed a pro-inflammatory diet in participants at their maximum weight at baseline conversely to WLM, which reported an anti-inflammatory dietary pattern regardless of the amount of weight loss. Notwithstanding, the group at their maximum weight was the only group capable of reducing their DII reporting a more anti-inflammatory dietary pattern after one year compared to baseline, as opposed to the other two groups which tend to increase the DII (change: maximum weight: −0.09; moderate WLM: 0.09; large WLM: 0.03).

## 4. Discussion

WLM reached their maximum weight at an earlier age than those entering the study at their maximum weight. Literature has related an earlier onset of obesity to more successful weight loss maintenance [27,28]. In the current study, those entering the study at their maximum weight lost more weight in the first year than WLM. Previous weight loss attempts were related to higher weight losses in the first year [9,27,29]; however, higher weight losses were also related to lower weight loss previously achieved [29]. On the one hand, Marinilli-Pinto et al. [30] described similar caloric intakes on the weight maintenance phase, regardless of weight loss previously achieved and method used. On the other hand, according to our results, successful weight losers have been related to lower spontaneous calorie intake [8]. Moreover, when a program for long term weight loss management was performed, caloric intake was similarly reduced after 6 months regardless of the previous weight-loss method [30]. One-year weight regain has been related to increases in calorie intake but also to higher baseline total energy intake [31]. More time will be needed to evaluate weight regain in our participants.

Our dietary intervention advices a high amount of healthful dietary fat [7] (such as nuts, olive oil, blue fish, etc.) in the context of a healthy Mediterranean diet, according to Predimed study findings [3]. That could explain the observed increase in all our groups. In studies like the current, fat intake was unaltered through time for all weight loss groups [30] and was similar regardless of the time they have maintained weight loss [32]. Julibert et al. [33] described in the current population that highest intakes of PUFA and MUFA were related to highest intakes of nuts and olive oil. Therefore, the highest increases in PUFA and MUFA intakes observed in the group reducing their BMI category before enrolment could be mediated by similar changes registered in nut consumption [34]. Bearing in mind that a reduction in caloric intake and an increase in fat intake were desirable due to the intervention applied [7], the reduction in calories happened at the expense of a reduction in carbohydrate intake.

The group at their maximum weight had the poorest dietary pattern at baseline (lowest MedDiet adherence, the only pro-inflammatory DII [18], and the highest unhealthful provegetarian pattern). Due to the dietary advice provided, similar MedDiet adherences were reported at one year, as previously established [4]. Average adherence to MedDiet improved in all groups from medium to high adherence [4], meaning that treatment was effective to increase MedDiet adherence. However, DII and unhealthful provegetarian pattern were improved after one year only in the group entering the study at their maximum weight. 

Even though caloric intake was lower in the group reducing their BMI category before enrolment, nutrient intake is not lower than in the other groups. This could be related to a lower intake of “empty” calories [35,36]. Moreover, previous research in our population has linked higher MedDiet adherence to an improvement in nutrient density after one year of intervention [4]. The higher improvement in MedDiet adherence among the group at their maximum weight might be mediated by the greater increase in consumption of white meat, vegetables and fruits described in that group, even when they registered the lowest intakes at baseline. Fruits (natural or dried) are sources of vitamin A, C, folate, magnesium, and potassium, while vegetables, especially green leafy vegetables, are sources of vitamins A, E, C, B2, and B6; folate; calcium; magnesium; and iron. For its part, white meat (mainly poultry) is a dietary source of vitamins B3, B6, B12, magnesium; phosphorus; and zinc [37,38]. This explains why intake of minerals and most vitamins changed similarly to vegetables, white meat, and fruits intake over the studied period. Dairy are sources of vitamin A, B2, B12; calcium; magnesium; and phosphorus; and when fortified, also of vitamin D [37,38]. Dairy intake was reduced in all groups after one year; however, the group at their maximum weight had lower decreases than the other groups. This, to some extent, might have helped them to maintain micronutrient intake at one year. Vitamin E contrasts with other nutrients. The group reducing their BMI category prior entering the study registered the highest increase in vitamin E intake, which can be related to nuts intake, as nuts are sources of vitamin E [37,38].

WLM, compared to weight-stable obese individuals, have healthier eating habits and are engaged in healthier diets [39]. On the one hand, previous research has linked spontaneous dieters to a higher BMI fluctuation [40], which could be related to repeated cycles of intentional weight loss. Conversely, unintentional weight loss was related to lesser physical activity, less concerns about dietary habits and lower lifetime maximum BMI [10]. Hence, it can be assumed that the group managing to reduce their BMI category might have less unintentional weight losers than the other groups. On the other hand, a study found that lifestyle interventions applied repeatedly in motivated people can be a method to maintain weight loss [41]. This could be the case for WLM. The proportion of hyperglycemia was higher in participants who had been in a higher BMI category prior to the inclusion. In Spain, the public health care system provides free dietary advice for diabetics [42], which could be a previous intervention received by the participants. This might be one of the causes why the group who decreased their BMI category had better dietary habits at baseline. In the current population, Bibiloni et al. [43] described that diabetic participants had overall better nutrient adequacy and MedDiet adherence compared to pre-diabetic or non-diabetic. Moreover, significances in adherence to 17-item MedDiet are lost after adjustment by T2DM prevalence at baseline. According to our results, history of dieting, especially with assistance, was inversely related to weight loss during a weight loss program [27,29] but also with lower weight regain [27]. Hence, history of DMT2 might lower the changes in adherence to the MedDiet, which could be related to a preceding history of dieting or to the free dietary advice that diabetics might have received.

Physical activity is an important and useful weight loss management tool, and it is widely used [11]. According to our results, the literature has described that successful WLM had spontaneously higher levels of overall physical activity at baseline [8,44] due to moderate physical activity [8,32]. However, no differences were found at one year in physical activity levels.

Previous research described that number of all kinds of weight loss strategies used, number of food related strategies, and keeping diet or exercise records diminish with time after weight loss [32]. Accordingly, the effort and the attention needed to maintain weight loss and dieting decreased through time [8,32]. Those changes in lifestyle should become habits as the literature has related weight regain to failure in maintaining those habits or behaviors [45]. Those may be reasons why the group that reduced their BMI category could focus on some specific items to improve it, because at baseline they already met more recommendations than the other groups. On the other hand, the group entering the study at their maximum weight was the only group able to improve dietary quality when it was measured by scales unrelated to intervention. This could have happened because their dietary quality at baseline was lower and their habits were not as stablished as in the other groups.

### Strengths and Limitations of the Study

To our knowledge, there is scarce scientific literature tackling dietary changes according to maximum weight or weight history in older adults. The current study increases the evidence on this topic and population. Other strengths of the present study are the longitudinal design that allows researchers to evaluate causality, the large sample size, and the standardized protocol that reduces the risk of information bias. Furthermore, the “maximum weight” groups that were made are very intuitive, as participants were classified according to clinical criteria. This classification is clinically relevant and easily calculable, and therefore, it enables transferring results to everyday clinical practice. Nonetheless, the present study has some limitations. Our main limitation is the absence of information regarding weight loss techniques previously used by participants. Moreover, little information about the anamnesis of the study participants and their weight history is available. Secondly, even though the study has two intervention groups, we worked with the study population as one cohort. Several food patterns were considered, one related and two unrelated to the intervention protocol, which allowed researchers to evaluate dietary quality regardless of the intervention group. Moreover, all analyses were adjusted by intervention group, to avoid confounding factors. Thirdly, despite the fact that the FFQ is a questionnaire validated in our population [14] and that it is a widely used method to evaluate food intake, there are other methods available that could be more appropriate to assess micronutrient intake [46]. Moreover, self-reported questionnaires are subject to some degree of information bias. With the intention to avoid bias, participants reporting extreme total energy intakes were excluded from the analysis [17]. Furthermore, the relationship of all parameters may represent association and/or causality. Lastly, all participants in the present study are adults over 55 years old at cardiovascular risk. Moreover, the lifestyle intervention applied to participants was specifically designed to avoid hard cardiovascular events [7]. Those are limitations to extend results to general adult population or to younger overweight adults, and to all kinds of weight management treatments.

## 5. Conclusions

The present study showed that in overweight population over 55 years, participants entering the study at their maximum weight were the most susceptible to improve significantly their dietary quality, assessed by adherence to Mediterranean diet, DII, and both healthful and unhealthful provegetarian patterns. People at their maximum weight might be the most benefitted in the short term by a weight management program. Long term weight loss efforts, while they are related to better spontaneous dietary patterns, may also reduce the effect of a weight management program. Presence of T2DM and history of dieting ought to be considered by health care professionals when suggesting a personalized weight loss strategy.

## Figures and Tables

**Table 1:** Baseline characteristics according to maximum weight and BMI at baseline.

	Current = Max ^§^ (*n* = 2181)	Moderate WLM ^§^ (*n* = 1688)	Large WLM ^§^ (*n* = 1826)	*p*-Value
	Mean (SD)	Mean (SD)	Mean (SD)	
Basal age (years)	65.1 (4.9)	65.1 (4.9)	65.0 (4.9)	n.s.
Basal BMI (kg/m^2^)	32.8 (3.5) ^a^	32.0 (3.0) ^a,c^	32.6 (3.7) ^c^	<0.001
maximum BMI (kg/m^2^)	32.6 (3.6) ^a,b^	33.7 (3.2) ^a,c^	36.2 (4.3) ^b,c^	<0.001
Difference basal vs. maximum BMI (kg/m^2^)	−0.1 (1.5) ^a^	1.7 (0.8) ^a,c^	3.5 (2.4) ^c^	<0.001
Perceived basal BMI (kg/m^2^)	32.6 (3.9) ^a^	32.1 (3.3) ^a,c^	32.7 (3.8) ^c^	<0.001
Reported ideal BMI (kg/m^2^)	27.1 (7.4)	29.4 (87.6)	27.7 (2.5)	n.s.
Age maximum BMI (years)	61.6 (9.1) ^a,b^	58.0 (9.8) ^a,c^	55.0 (11.4) ^b,c^	<0.001
	*n* (%)	*n* (%)	*n* (%)	
Sex (female)	1149 (52.7)	705 (41.8)	893 (48.9)	<0.001
Intervention group (energy reduced MedDiet)	1074 (49.2)	906 (53.7)	910 (49.8)	0.019
Education level				
Primary	1028 (47.5)	816 (48.8)	954 (52.7)	0.005
Secondary	634 (29.3)	485 (29.0)	512 (28.3)	
Tertiary	502 (23.2)	372 (22.2)	343 (19.0)	
Marital status				
Married	1646 (75.6)	1351 (80.3)	1374 (75.6)	0.004
Divorced/separated	160 (7.4)	118 (7.0)	142 (7.8)	
Widower	239 (11.0)	149 (8.9)	209 (11.5)	
Other (single + religious)	131 (6.0)	65 (3.9)	92 (5.1)	
Living alone ‡	293 (13.4)	166 (9.8)	235 (12.9)	0.002
Smoking habit				
Current smoker	248 (11.4)	217 (12.9)	234 (12.9)	n.s.
Former smoker	935 (43.0)	750 (44.5)	761 (42.0)	
Never smoked	989 (45.5)	717 (42.6)	818 (45.1)	
MetS components				
High blood pressure	2000 (91.7)	1549 (91.8)	1688 (92.4)	n.s.
Hyperglycemia	1570 (72.0)	1270 (75.2)	1462 (80.1)	<0.001
Hypertriglyceridemia	1263 (57.9)	952 (56.4)	974 (53.3)	0.014
Low HDL-cholesterol	895 (41.0)	722 (42.8)	817 (44.7)	n.s.
Abdominal obesity	2099 (96.2)	1626 (96.3)	1741 (95.3)	n.s.

Abbreviations: Max: Maximum. SD: Standard deviation. BMI: Body Mass Index. MedDiet: Mediterranean Diet. HDL-cholesterol: High density lipoprotein-cholesterol. n.s.: non statistically significant. ^§^ Difference between maximum and current BMI at baseline (maximum weight − current weight (baseline)): Current = Max: baseline current weight is their maximum weight. Moderate WLM: participants who lost weight within the same BMI category. Large WLM: participants who lost weight and decrease at least one BMI category. ‡ Living alone regardless of marital status. ^a,b,c^ Different letters show differences between groups: Differences in means between groups were tested by one-way ANOVA and Bonferroni’s post hoc; differences in prevalence’s across groups were examined using χ^2^.

**Table 2:** Dietary patterns, physical activity pattern, and BMI changes according to maximum weight and BMI at baseline.

		Current = Max ^§^ (*n* = 2181)	Moderate WLM ^§^ (*n* = 1688)	Large WLM ^§^ (*n* = 1826)	Time*group ‡
		Mean (SD)	Mean (SD)	Mean (SD)	
Energy	Baseline	2390.4 (548.9) ^b^	2395.1 (546.5) ^c^	2319.5 (548.6) ^b,c^	n.s.
(kcal/d)	1 year	2251.2 (471.6) ^b^	2271.8 (477.0) ^c^	2210.9 (474.7) ^b,c^	
	∆	−139.1 (528.8) *	−123.3 (555.5) *	−108.6 (525.6) *	
DII ‡	Baseline	0.11 (2.0) ^a,b^	−0.07 (2.0) ^a^	−0.07 (2.0) ^b^	<0.001
	1 year	0.02 (2.0)	0.02 (2.1)	−0.04 (2.0)	
	∆	−0.09 (2.1) *^,d,e^	0.09 (2.1) ^d^	0.03 (2.1) ^e^	
Healthful	Baseline	53.7 (6.5)	54.1 (6.5)	53.9 (6.5)	n.s.
provegetarian	1 year	53.6 (7.0)	54.0 (7.0)	53.9 (7.1)	
pattern	∆	−0.1 (7.8)	−0.1 (7.7)	0.0 (7.8)	
Unhealthful	Baseline	54.7 (6.9) ^a,b^	53.6 (7.1) ^a,c^	53.2 (7.1) ^b,c^	0.013
provegetarian	1 year	54.0 (7.6) ^a,b^	54.0 (7.4) ^a^	53.4 (7.2) ^b^	
pattern	∆	−0.7 (8.3) *^,e^	0.3 (8.5)	0.2 (8.1) ^e^	
17 items	Baseline	8.3 (2.6) ^a,b^	8.5 (2.8) ^a^	8.7 (2.6) ^b^	0.046^#^
MedDiet ⁂	1 year	11.7 (3.0)	11.7 (2.9)	11.8 (2.8)	
	∆	3.4 (3.3) *^,e,#^	3.2 (3.4) *	3.1 (3.2) *^,e,#^	
Light PA	Baseline	766.2 (940.3)	759.5 (936.1)	777.8 (971.7)	n.s.
(METs) †	1 year	803.2 (3785.8)	827.6 (959.1)	826.2 (965.7)	
	∆	37.0 (3841.2)	68.1 (1134.2) *	48.4 (1102.5)	
Moderate PA	Baseline	872.0 (1370.4) ^b^	1029.7 (1622.7)	1050.9 (1698.5) ^b^	n.s.
(METs) †	1 year	1148.2 (3997.7)	1289.9 (1797.5)	1278.8 (1787.2)	
	∆	276.2 (3979.7) *	260.3 (1746.5) *	227.9 (1724.7) *	
Intense PA	Baseline	729.6 (1275.2)	806.2 (1500.8)	788.4 (1541.6)	n.s.
(METs) †	1 year	1027.1 (4176.6)	984.4 (1660.1)	1007.9 (1662.9)	
	∆	297.5 (4186.0) *	178.2 (1615.9) *	219.5 (1663.7) *	
Total PA	Baseline	2367.8 (2142.1) ^b^	2595.4 (2408.8)	2617.1 (2457.1) ^b^	n.s.
(METs) †	1 year	2978.5 (4473.4)	3101.9 (2531.5)	3112.9 (2559.4)	
	∆	610.7 (4476.8) *	506.5 (2552.4) *	495.8 (2428.8) *	
Chair-test Ⱡ	Baseline	13.3 (4.9)	13.5 (4.9)	13.2 (4.9)	n.s.
	1 year	14.2 (6.1)	14.2 (6.0)	13.8 (6.2)	
	∆	0.9 (5.6) *	0.7 (5.4) *	0.7 (5.5) *	
BMI (kg/m^2^)	Baseline	32.8 (3.5) ^a^	32.0 (3.0) ^a,c^	32.6 (3.7) ^c^	<0.001
	1 year	31.7 (3.7) ^a^	31.2 (3.3) ^a,c^	31.9 (3.9) ^c^	
	∆	−1.0 (1.5) *^,d,e^	−0.8 (1.5) *^,d,f^	−0.7 (1.7) *^,e,f^	

**Abbreviations**: Max: Maximum. Categ: Category. SD: Standard deviation. BMI: Body Mass Index. ∆: Change between baseline and 1 year. DII: Dietary inflammatory index. 17 item MedDiet: 17-item Mediterranean dietary questionnaire. BMI: Body Mass Index. n.s.: non statistically significant. PA: Physical Activity. † Measured in MET (Metabolic equivalent of task) min/week; 3 subjects were excluded from the analysis due to missing data. ^§^ Difference between maximum and current BMI at baseline (maximum weight − current weight (baseline)): Current = Max: baseline current weight is their maximum weight. Moderate WLM: participants who lost weight within the same BMI category. Large WLM: participants who lost weight and decrease at least one BMI category. ⁂ 3 subjects were excluded from the analysis due to missing data. Ⱡ 97 subjects were excluded from the analysis due to missing data. ‡ Data analyzed by two-way repeated measures ANCOVA adjusted by gender and randomization. *p* < 0.05. ‡ DII analysis was also adjusted by energy intake and physical activity. Different letters indicate statistically significant differences between groups (^a,b,c^), between time (*) and between time*group interaction (^d,e,f^) by the Bonferroni post hoc test (*p* < 0.05). ^#^ Time*group significances lost after adjustment by presence of Diabetes Mellitus 2 at baseline.

## Supplementary Materials

The following are available online at https://www.mdpi.com/2072-6643/12/10/3023/s1, Table S1: Nutritional density at baseline and 1-year follow-up according to maximum weight and BMI at baseline, Table S2: Food intake (dietary items; g/d) at baseline and 1-year follow- up according to maximum weight and BMI at baseline.
