# Supplementary material for: Dietary Quality Changes According to the Preceding Maximum Weight: A Longitudinal Analysis in the PREDIMED-Plus Randomized Trial
Source: Nutrients. 2020 Oct 2;12(10):3023. doi: 10.3390/nu12103023 (PMC7600377; doi:10.3390/nu12103023)
Supplement: Supplementary file 1 [file nutrients-12-03023-s001.pdf]

Supplementary table 1. Nutritional density at baseline and 1-year follow-up according to maximum weight and BMI at baseline.

		**Current = Max§ (*n* =2181)**	**moderate WLM§ (*n* =1688)**	**large WLM§ (*n* =1826)**	***Time* group* ‡**
		Mean (SD)	Mean (SD)	Mean (SD)	
Carbohydrates (g/1000kcal)	Baseline	100.6 (17.2) [b]	101.1 (16.7)	101.9 (17.3) [b]	n.s.
	1 year	93.4 (15.0)	93.8 (14.5)	94.2 (15.2)	
	Δ	-7.3 (17.4)*	-7.3 (17.3)*	-7.7 (17.0)*	
Proteins (g/1000kcal)	Baseline	41.3 (6.8)[a,b]	41.6 (6.9)[a,c]	43.0 (7.3)[b,c]	<0.001
	1 year	43.1 (6.8) [b]	42.8 (6.6) [c]	43.8 (6.8)[b,c]	
	Δ	1.8 (7.1)*[d,e]	1.2 (7.2)* [d]	0.8 (7.4)* [e]	
Lipids (g/1000kcal)	Baseline	44.4 (7.2) [b]	44.0 (7.2)	43.7 (7.3) [b]	n.s.
	1 year	47.0 (6.4)	46.8 (6.4)	46.9 (6.5)	
	Δ	2.7 (7.7)*	2.9 (7.8)*	3.2 (7.9)*	
PUFA (g/1000kcal)	Baseline	7.1 (2.0)	7.1 (2.1)	7.0 (2.0)	0.010
	1 year	8.2 (1.9)	8.2 (1.9)	8.3 (1.9)	
	Δ	1.2 (2.3)*	1.0 (2.5)* [f]	1.3 (2.4)* [f]	
MUFA (g/1000kcal)	Baseline	23.1 (5.1) [b]	22.9 (5.3)	22.6 (5.1) [b]	0.008
	1 year	26.7 (4.9)	26.6 (5.1)	26.8 (5.2)	
	Δ	3.6 (6.0)* [e]	3.7 (6.2)*	4.2 (6.1)* [e]	
SFA (g/1000kcal)	Baseline	11.1 (2.2)	11.0 (2.2)	11.0 (2.2)	n.s.
	1 year	10.2 (1.9) [b]	10.3 (1.9)	10.1 (1.8) [b]	
	Δ	-0.9 (2.2)*	-0.7 (2.2)*	-0.9 (2.3)*	
Trans Fat (g/1000kcal)	Baseline	0.2 (0.1) [b]	0.2 (0.1)	0.2 (0.1) [b]	n.s.
	1 year	0.2 (0.1)	0.2 (0.1)	0.2 (0.1)	
	Δ	-0.1 (0.1)*	-0.1 (0.1)*	-0.1 (0.1)*	
Cholesterol (mg/1000kcal)	Baseline	161.8 (41.5) [b]	162.9 (43.6) [c]	166.9 (42.4)[b,c]	0.010
	1 year	159.0 (38.1)	158.2 (37.3)	159.6 (41.4)	
	Δ	-2.7 (46.6)* [e]	-4.7 (47.6)*	-7.3 (48.9)* [e]	
Fiber (g/1000kcal)	Baseline	10.9 (3.4)[a,b]	11.2 (3.5)[a,c]	11.7 (3.6)[b,c]	0.017
	1 year	13.4 (3.5)[a,b]	13.5 (3.4)[a,c]	13.9 (3.6)[b,c]	
	Δ	2.5 (3.7)* [e]	2.2 (3.7)*	2.2 (3.7)* [e]	
Vitamin A (mcg/1000kcal)	Baseline	457.8 (267.3)[a,b]	482.7 (280.2) [a]	496.8 (281.2) [b]	0.003
	1 year	470.9 (257.3)	467.4 (222.7)	482.0 (243.9)	
	Δ	13.2 (304.5)*[d,e]	-15.3 (297.1)* [d]	-14.8 (300.8)* [e]	
Vitamin B1 (mg/1000kcal)	Baseline	0.7 (0.1)[a,b]	0.7 (0.1)[a,c]	0.7 (0.1)[b,c]	0.010
	1 year	0.7 (0.1) [b]	0.7 (0.1) [c]	0.8 (0.1)[b,c]	
	Δ	0.1 (0.1)* [e]	0.0 (0.1)*	0.0 (0.1)* [e]	
Vitamin B2 (mg/1000kcal)	Baseline	0.9 (0.3)[a,b]	0.9 (0.3)[a,c]	0.9 (0.3)[b,c]	n.s.
	1 year	0.9 (0.3) [b]	0.9 (0.3) [c]	0.9 (0.3)[b,c]	
	Δ	0.1 (0.3)*	0.0 (0.3)*	0.0 (0.2)*	
Vitamin B3 (mg/1000kcal)	Baseline	17.3 (3.7)[a,b]	17.4 (3.7)[a,c]	18.0 (3.9)[b,c]	<0.001
	1 year	18.6 (3.7) [b]	18.3 (3.4) [c]	18.8 (3.7)[b,c]	
	Δ	1.3 (3.9)*[d,e]	0.9 (3.8)* [d]	0.8 (4.0)* [e]	
Vitamin B6 (mg/1000kcal)	Baseline	1.0 (0.2)[a,b]	1.0 (0.2)[a,c]	1.0 (0.2)[b,c]	0.001
	1 year	1.1 (0.2) [b]	1.1 (0.2) [c]	1.1 (0.2)[b,c]	
	Δ	0.1 (0.2)*[d,e]	0.1 (0.2)* [d]	0.1 (0.2)* [e]	
Vitamin B12 (mcg/1000kcal)	Baseline	4.2 (1.8)[a,b]	4.3 (1.9) [a]	4.4 (1.9) [b]	0.001
	1 year	4.5 (1.9)	4.4 (1.6)	4.5 (1.7)	
	Δ	0.3 (2.0)*[d,e]	0.1 (2.1) [d]	0.1 (2.0) [e]	
Vitamin B9	Baseline	148.4 (40.3)[a,b]	151.8 (42.6) [a]	156.3 (42.7) [b]	<0.001

(Folic acid)	1 year	170.6 (41.8) [b]	169.3 (39.6)	173.8 (41.1) [b]	
(mcg/1000kcal)	Δ	22.2 (43.4)*[d,e]	17.4 (43.7)* [d]	17.5 (43.3)* [e]	
Vitamin C	Baseline	85.5 (36.4)[a,b]	86.9 (37.4)[b,c]	91.7 (37.7)[a,c]	<0.001
(mg/1000kcal)	1 year	98.4 (37.5)	96.8 (34.8)	100.0 (35.8)	
	Δ	12.9 (39.2)*[d,e]	9.9 (37.9)* [d]	8.3 (37.5)* [e]	
Vitamin D	Baseline	2,6 (1,5) [b]	2,7 (1,5)	2.7 (1.5) [b]	n.s.
(mcg/1000kcal)	1 year	3,2 (1,6)	3,1 (1,5)	3.1 (1.7)	
	Δ	0,5 (1,8)*	0,4 (1,8)*	0.4 (1.9)*	
Vitamin E	Baseline	4,4 (1,3)[a,b]	4,6 (1,4) [a]	4.5 (1.3) [b]	0.034
(mg/1000kcal)	1 year	5,3 (1,3) [b]	5,3 (1,3)	5.4 (1.3) [b]	
	Δ	0.9 (1.5)*	0.7 (1.7)* [f]	0.9 (1.6)* [f]	
Calcium (Ca)	Baseline	433.5 (128.7)[a,b]	440.6 (128.3)[a,c]	456.2 (137.2)[b,c]	0.003
(mg/1000kcal)	1 year	445.4 (121.9)[a,b]	448.8 (122.6) [a]	454.3 (124.1) [b]	
	Δ	11.9 (130.2)* [e]	8.3 (127.3)* [f]	-2.0 (134.4)[e,f]	
Magnesium	Baseline	176.4 (35.1)[a,b]	179.7 (35.5)[a,c]	184.6 (36.7)[b,c]	0.042
(Mg)	1 year	204.1 (37.0)[a,b]	204.1 (37.0)[a,c]	209.8 (37.1)[b,c]	
(mg/1000kcal)	Δ	27.7 (39.2)*	24.4 (39.2)*	25.2 (39.2)*	
Phosphorus (P)	Baseline	741.4 (142.8)[a,b]	749.1 (142.1)[a,c]	780.0 (152.5)[b,c]	<0.001
(mg/1000kcal)	1 year	803.7 (144.9) [b]	797.8 (141.2) [c]	822.2 (145.6)[b,c]	
	Δ	62.3 (144.4)*[d,e]	48.7 (143.7)* [d]	42.2 (146.9)* [e]	
Iron (Fe)	Baseline	6.9 (1.2)[a,b]	7.0 (1.2)[a,c]	7.2 (1.2)[b,c]	0.005
(mg/1000kcal)	1 year	7.5 (1.2) [b]	7.5 (1.2) [c]	7.6 (1.2)[b,c]	
	Δ	0.5 (1.3)* [e]	0.4 (1.3)*	0.4 (1.3)* [e]	
Selenium (Se)	Baseline	49.5 (10.4) [b]	49.5 (10.7) [c]	51.2 (10.9)[b,c]	<0.001
(mcg/1000kcal)	1 year	51.1 (10.3)	50.4 (9.8)	51.4 (10.4)	
	Δ	1.6 (11.5)* [e]	0.9 (11.3)*	0.2 (12.4) [e]	
Zinc (Zn)	Baseline	5.6 (0.9) [b]	5.6 (0.9) [c]	5.8 (1.0)[b,c]	0.002
(mg/1000kcal)	1 year	5.7 (0.9) [b]	5.7 (0.9) [c]	5.8 (0.9)[b,c]	
	Δ	0.2 (1.0)* [e]	0.1 (1.0)*	0.1 (1.1)* [e]	
Iodine (I)	Baseline	118.8 (66.2)[a,b]	121.7 (67.5)[a,c]	129.1 (71.1)[b,c]	0.026
(mcg/1000kcal)	1 year	122.7 (64.2)[a,b]	124.8 (65.7) [a]	127.7 (66.2) [b]	
	Δ	3.9 (64.8)* [e]	3.1 (66.8)*	-1.4 (66.4) [e]	
Potassium (K)	Baseline	1887.7 (395.3)[a,b]	1925.4 (409.5)[a,c]	1984.6 (415.3)[b,c]	<0.001
(g/1000kcal)	1 year	2117.5 (402.2) [b]	2105.5 (386.0) [c]	2159.5 (388.3)[b,c]	
	Δ	229.8 (417.0)*[d,e]	180.1 (428.1)* [d]	174.9 (418.4)* [e]	

Abbreviations: Max: Maximum. SD: Standard deviation. BMI: Body Mass Index. Δ: Change between baseline and 1 year. PUFA: Polyunsaturated fatty acids. MUFA: Monounsaturated fatty acids. SFA: Saturated fatty acids. n.s.: non statistically significant. §Difference between maximum and current BMI at baseline [maximum weight - current weight (baseline)]. 1) Current = Max: baseline current weight is their maximum weight. 2) Moderate WLM: participants who lost weight within the same BMI category. 3) Large WLM: participants who lost weight and decrease at least one BMI category. ‡Data analyzed by two-way repeated measures ANCOVA adjusted by gender and randomization. p<0.05. Different letters indicate statistically significant differences between groups (a, b, c), between time (*) and between time*group interaction (d, e, f) by the Bonferroni post-hoc test (p<0.05).

Supplementary table 2. Food intake (dietary items; g/d) at baseline and 1-year follow-up according to maximum weight and BMI at baseline.

		**Current = Max[§] (*n* =2181)**	**moderate WLM[§] (*n* =1688)**	**large WLM[§] (*n* =1826)**	***Time* group* ‡**
		Mean (SD)	Mean (SD)	Mean (SD)	
Fruits (g/d)	Baseline	392.0 (225.7)[a,b]	411.2 (230.6) [a]	408.3 (227.3) [b]	0.026 [#]
	1 year	444.3 (217.2)	450.1 (210.3)	446.8 (209.1)	
	Δ	52.3 (254.7)* [e #]	38.9 (245.7)*	38.5 (238.2)* [e#]	
Vegetables (g/d)	Baseline	322.7 (135.4)[a,b]	330.8 (140.3) [a]	338.7 (137.5) [b]	0.001
	1 year	364.7 (141.6)	363.2 (138.6)	366.0 (139.1)	
	Δ	41.9 (153.7)* [e]	32.4 (154.3)*	27.4 (147.2)* [e]	
Potatoes (g/d)	Baseline	68.2 (43.8)	69.1 (44.3)	66.4 (44.4)	n.s.
	1 year	65.6 (42.4)	64.0 (39.4)	64.3 (40.2)	
	Δ	-2.7 (48.4)	-5.1 (48.1)*	-2.1 (49.1)*	
Refined cereals (g/d)	Baseline	112.9 (86.9)	112.1 (87.7)	104.3 (89.2)	n.s.
	1 year	67.2 (71.9)	67.6 (71.8)	61.7 (69.0)	
	Δ	-45.6 (90.5)*	-44.5 (92.9)*	-42.6 (91.0)*	
Whole grains (g/d)	Baseline	37.6 (63.3) [b]	36.3 (60.9) [c]	42.8 (62.8)[b,c]	n.s.
	1 year	60.1 (62.7)	60.4 (63.9)	63.5 (62.1)	
	Δ	22.5 (75.1)*	24.1 (73.0)*	20.6 (74.3)*	
Legumes (g/d)	Baseline	19.8 (11.0)[a,b]	21.1 (10.5) [a]	20.9 (11.3) [b]	n.s.
	1 year	24.0 (10.1)[a,b]	25.2 (11.2) [a]	25.3 (11.9) [b]	
	Δ	4.2 (12.7)*	4.1 (13.1)*	4.4 (14.2)*	
White fish (g/d)	Baseline	37.8 (25.9)	37.4 (25.1)	38.8 (24.8)	n.s.
	1 year	42.9 (24.7)	42.3 (24.4)	42.4 (25.9)	
	Δ	5.1 (29.1)*	5.0 (28.9)*	3.5 (29.6)*	
Blue fish (g/d)	Baseline	36.2 (22.8)	37.1 (23.2)	36.8 (23.1)	0.062
	1 year	42.8 (23.2)	42.5 (23.3)	41.8 (24.5)	
	Δ	6.6 (26.1)* [e #]	5.5 (27.6)*	5.0 (27.6)* [e #]	
Seafood (g/d)	Baseline	27.4 (21.3)	27.9 (21.0)	27.8 (22.0)	n.s.
	1 year	27.4 (22.1)	27.1 (20.2)	26.4 (21.3)	
	Δ	0.0 (25.1)	-0.8 (22.5)	-1.4 (24.0)*	
White meat (g/d)	Baseline	60.6 (33.3) [b]	61.9 (34.0)	64.0 (34.3) [b]	0.002
	1 year	68.9 (32.5)	67.0 (30.4)	69.3 (31.8)	
	Δ	8.2 (36.4)*[d,e]	5.1 (34.0)* [d]	5.3 (36.0)* [e]	
Red meat (g/d)	Baseline	52.4 (35.4) [a]	49.9 (33.7) [a]	49.3 (33.8)	n.s.
	1 year	36.4 (29.4)	36.6 (27.1)	35.2 (28.8)	
	Δ	-16.1 (36.9)*	-13.3 (32.8)*	-14.1 (34.1)*	
Processed meat (g/d)	Baseline	35.5 (24.3)	36.6 (24.4)	34.8 (23.5)	n.s.
	1 year	27.5 (17.3)	27.6 (17.3)	27.4 (17.6)	
	Δ	-8.0 (24.3)*	-9.0 (24.7)*	-7.4 (24.5)*	
Eggs (g/d)	Baseline	23.7 (11.6)	23.9 (11.3)	24.2 (11.8)	n.s.
	1 year	24.6 (10.3)	24.9 (10.4)	24.7 (11.0)	
	Δ	0.9 (12.1)*	1.0 (12.7)*	0.5 (12.2)	
Milk and dairy (g/d)	Baseline	334.4 (193.3) [b]	341.8 (202.3) [c]	356.6 (206.9)[b,c]	0.014
	1 year	323.3 (190.1) [b]	329.0 (193.0)	332.9 (193.0) [b]	
	Δ	-11.1 (183.1)* [e]	-12.8 (196.4)*	-23.7 (193.1)* [e]	
Nuts (g/d)	Baseline	15.1 (18.0)	15.6 (17.2)	14.4 (16.1)	0.002
	1 year	28.5 (17.8) [b]	28.8 (19.3)	30.4 (19.4) [b]	
	Δ	13.3 (22.1)* [e]	13.2 (23.2)* [f]	15.9 (22.0)*[e,f]	
Olive oil (g/d)	Baseline	41.3 (16.6) [b]	40.1 (16.8) [c]	38.9 (16.9)[b,c]	n.s.
	1 year	46.5 (14.9) [b]	46.0 (14.9)	44.7 (15.5) [b]	

	Δ	5.2 (19.3)*	5.9 (19.6)*	5.8 (19.7)*	
Vegetal oils (g/d)	Baseline	1.4 (5.0)	1.7 (5.7)	1.3 (5.1)	n.s.
	1 year	0.5 (3.2)	0.6 (3.4)	0.5 (2.3)	
	Δ	-0.8 (5.1)*	-1.1 (6.2)*	-0.9 (5.4)*	
Other fats (g/d)	Baseline	2.7 (6.0)	2.6 (8.0)	2.4 (5.7)	n.s.
	1 year	1.0 (3.1)	1.2 (7.1)	1.0 (3.0)	
	Δ	-1.7 (5.8)*	-1.4 (5.4)*	-1.5 (5.8)*	
Sweets and pastries (g/d)	Baseline	47.4 (36.9)	47.3 (39.4)	44.4 (39.9)	n.s.
	1 year	28.7 (28.8)	29.1 (29.5)	27.4 (27.8)	
	Δ	-18.7 (37.4)*	-18.2 (39.6)*	-17.0 (38.4)*	
Convenience foods (g/d)	Baseline	22.2 (20.5)	22.7 (22.7)	20.6 (19.6)	n.s.
	1 year	16.7 (16.2)	17.8 (17.9) [c]	15.7 (16.4) [c]	
	Δ	-5.6 (20.0)*	-4.9 (22.0)*	-4.9 (19.4)*	
Coffee and tea (ml/d)	Baseline	86.9 (58.6)	90.2 (60.3)	88.4 (60.1)	n.s.
	1 year	86.3 (59.0)	87.9 (60.2)	88.7 (62.4)	
	Δ	-0.7 (56.6)	-2.3 (57.8)	0.3 (56.6)	
Sugary beverages (ml/d)	Baseline	40.0 (88.0)	39.9 (90.4)	38.1 (89.1)	n.s.
	1 year	19.6 (66.9)	20.5 (59.4)	20.6 (66.7)	
	Δ	-20.4 (100.7)*	-19.3 (93.8)*	-17.5 (91.3)*	
Artificially sweetened beverages(ml/d)	Baseline	28.4 (101.4)	26.1 (93.3)	33.8 (115.6)	n.s.
	1 year	23.0 (83.4)	20.1 (66.0)	25.2 (80.6)	
	Δ	-5.4 (90.4)*	-6.0 (77.0)*	-8.6 (97.1)*	
Fermented alcoholic beverages(ml/d)	Baseline	186.0 (266.3) [b]	186.6 (261.9)	149.3 (244.9) [b]	n.s.
	1 year	154.6 (230.8) [b]	158.5 (234.3)	128.3 (220.8) [b]	
	Δ	-31.3 (203.2)*	-28.1 (218.0)*	-21.1 (192)*	
Distilled spirits (ml/d)	Baseline	3.5 (11.6)	3.8 (11.4)	3.0 (9.2)	n.s.
	1 year	2.2 (9.5)	2.9 (11.5)	2.2 (7.8)	
	Δ	-1.3 (11.6)*	-0.9 (11.9)*	-0.9 (8.2)*	

Abbreviations: Max: Max: Maximum. SD: Standard deviation. BMI: Body Mass Index: Δ. Change between baseline and 1 year. n.s.: non statistically significant. §Difference between maximum and current BMI at baseline [maximum weight - current weight (baseline)]. 1) Current = Max: baseline current weight is their maximum weight. 2) Moderate WLM: participants who lost weight within the same BMI category. 3) Large WLM: participants who lost weight and decrease at least one BMI category. ‡Data analyzed by two-way repeated measures ANCOVA adjusted by gender, randomization, energy intake and physical activity. $p<0.05$. Different letters indicate statistically significant differences between groups (a, b, c), between time (*) and between time*group interaction (d, e, f) by the Bonferroni post-hoc test ($p<0.05$). # Time*group significances lost after adjustment by presence of Type 2 Diabetes Mellitus at baseline.
